# A wearable, flexible sensor for real-time, home monitoring of sleep apnea

**DOI:** 10.1016/j.isci.2022.104163

**Published:** 2022-03-25

**Authors:** Satoko Honda, Hyuga Hara, Takayuki Arie, Seiji Akita, Kuniharu Takei

**Affiliations:** 1 Department of Physics and Electronics, Osaka Prefecture University, Sakai, Osaka 599-8531, Japan; 2 Department of Physics and Electronics, Osaka Metropolitan University, Sakai, Osaka 599-8531, Japan

**Keywords:** Clinical measurement in health technology, Bioelectronics, Materials in biotechnology

## Abstract

A flexible sensor that can be attached to the body to collect vital data wirelessly enables real-time human healthcare management. One potential application for home-use healthcare devices is monitoring of sleep conditions to diagnose sleep apnea syndrome. Such data are not readily gathered using conventional tools, owing to the bulk and cost of instrumentation. In order to monitor respiration at home, it is necessary to improve sensing performance and long-term stability of the sensors without sacrificing wearability and comfortability. To build a platform for wireless home-use respiration monitoring, this study develops a mask-borne flexible humidity sensor using ZnIn_2_S_4_ nanosheets as a humidity-sensitive material with high sensitivity and stability for more than 150 h. As proof-of-concept, long-term wireless respiration monitoring is demonstrated during sleep to identify symptoms of sleep apnea in wearers.

## Highlights

• A highly stable humidity sensor
• Wireless wearable respiration monitoring
• Per-form for sleep apnea monitoring

## Graphical abstract

**Figure:**
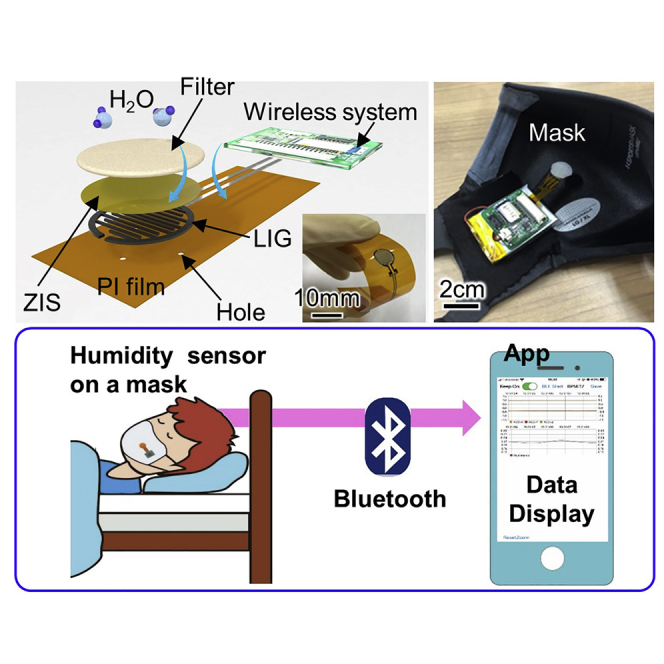

## Introduction

Continuous, real-time vital information allows detection of abnormal vital sign changes that may be caused by a disease. If such signs can be detected at an early stage, there is a greater chance of successful treatment and of saving a life. However, monitoring must measure continuous vital data during daily life in order to identify small changes. Because conventional medical instrumentation is unsuitable for home monitoring owing to bulky and expensive sensors, wearable sensors have the potential to revolutionize vital monitoring. Moreover, to enhance their wearability and comfort, wearable flexible sensors have been widely reported for healthcare applications (Bariya et al., 2018; Heikenfeld et al., 2018; Xu et al., 2019, 2021b). One important syndrome, which can be monitored at home by measuring respiration rate, is sleep apnea (Kwon et al., 2021). In fact, approaches for home-use respiration rate monitoring have been proposed using pressure sensors (Zhong et al., 2021), strain sensors (Xu et al., 2021a), accelerometers (Zavanelli et al., 2021), and humidity sensors (Lu et al., 2021b; Pang et al., 2018; Xie et al., 2019). As a flexible sensor system, a pressure or humidity sensor attached to a mask may be a useful platform for respiration monitoring (Escobedo et al., 2022; Pang et al., 2018; Zhong et al., 2021). For this purpose, accurate and reliable data detection during sleep is required in order to diagnose sleep quality precisely. Although some studies have reported a mask-borne flexible respiration sensor (Escobedo et al., 2022; Leelasree et al., 2020; Pang et al., 2018; Zhong et al., 2021), long-term monitoring during sleep employing a flexible sensor on a mask has yet to be demonstrated.

To overcome the challenge of employing a flexible sensor in a long-term practical application, this study demonstrates a highly stable, flexible humidity sensor with an integrated, wireless circuit system attached to a mask. Water molecules in breath are detected by a ZnIn_2_S_4_ (ZIS) nanosheet-based humidity sensor (Lu et al., 2020, 2021a). Using ZIS material, we have already reported a flexible humidity sensor for long-term, stable detection. This sensor shows rapid response and stable detection, mostly owing to inorganic, two-dimensional nanomaterials. However, sensor structure has not yet been optimized, with the result that stability of sensitivity is still impeding the design of such devices. To address this issue, this study first discusses design optimization to achieve high stability by changing the electrode distance and thickness of the ZIS stacking layer. After optimizing the design, a wireless system and a smart phone app are installed on a commercial mask to monitor real-time, continuous respiration during sleep. Finally, as proof-of-concept, respiration monitoring during sleep is demonstrated on three subjects.

## Results and discussions

### Humidity sensor optimizations

**Figure 1:**
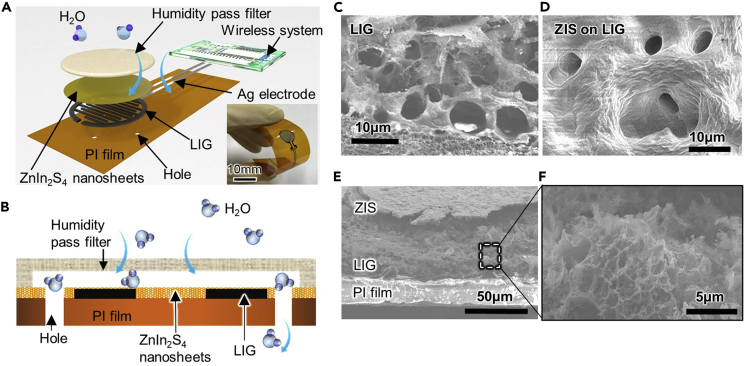
Flexible ZIS humidity sensor (A) Schematic illustration and photo of the flexible humidity sensor. (B) Cross-sectional schematic of the humidity sensor. SEM image of (C) LIG surface, (D) ZIS surface on LIG, (E) cross-sectional views of ZIS, LIG, and PI film layer, and (F) enlarged cross-sectional LIG.

The resistive flexible humidity sensor consists of a polyimide (PI) film, laser-induced graphene (LIG) electrodes, a ZIS stacking layer, and a humidity pass filter (Figure 1A). A wireless system with a battery measures a voltage change converted from a sensor resistance change, and resulting measurements are transmitted to a smart phone using Bluetooth. For the previous study, we concluded that LIG electrodes constitute a sensitive, stable interface for ZIS to measure humidity (Lu et al., 2020). The humidity pass filter was used to prevent water from making direct contact with the surface of the ZIS in order to prevent a malfunction. A few holes were punched in the PI film between the LIG interdigital electrodes to evacuate water molecules quickly from the ZIS surface to accomplish continuous humidity monitoring (Figure 1B).

For sensor components, ZIS and LIG are the main materials to detect humidity changes. Conductance of the ZIS nanosheet is changed by Fermi level movement between HOMO and LUMO caused by a proton-hopping process between H_3_O^+^ ions and water molecules (Lu et al., 2020). The atomic ratio of Zn, In, and S for ZIS is 1:1.8:2.8, suggesting that highly *c*-axis-oriented ZIS used in this study has sulfur vacancies and it is most likely substituted by oxygen atoms (Lu et al., 2020). LIG electrodes are porous structures (Figure 1C), and the surface morphology of the ZIS nanosheets shows wrinkled structure because of stacking of nanosheets in layers (Figure 1D). Furthermore, the ZIS nanosheets are uniformly stacked so as to cover the porous LIG electrodes (Figures 1E and 1F).

**Figure 2:**
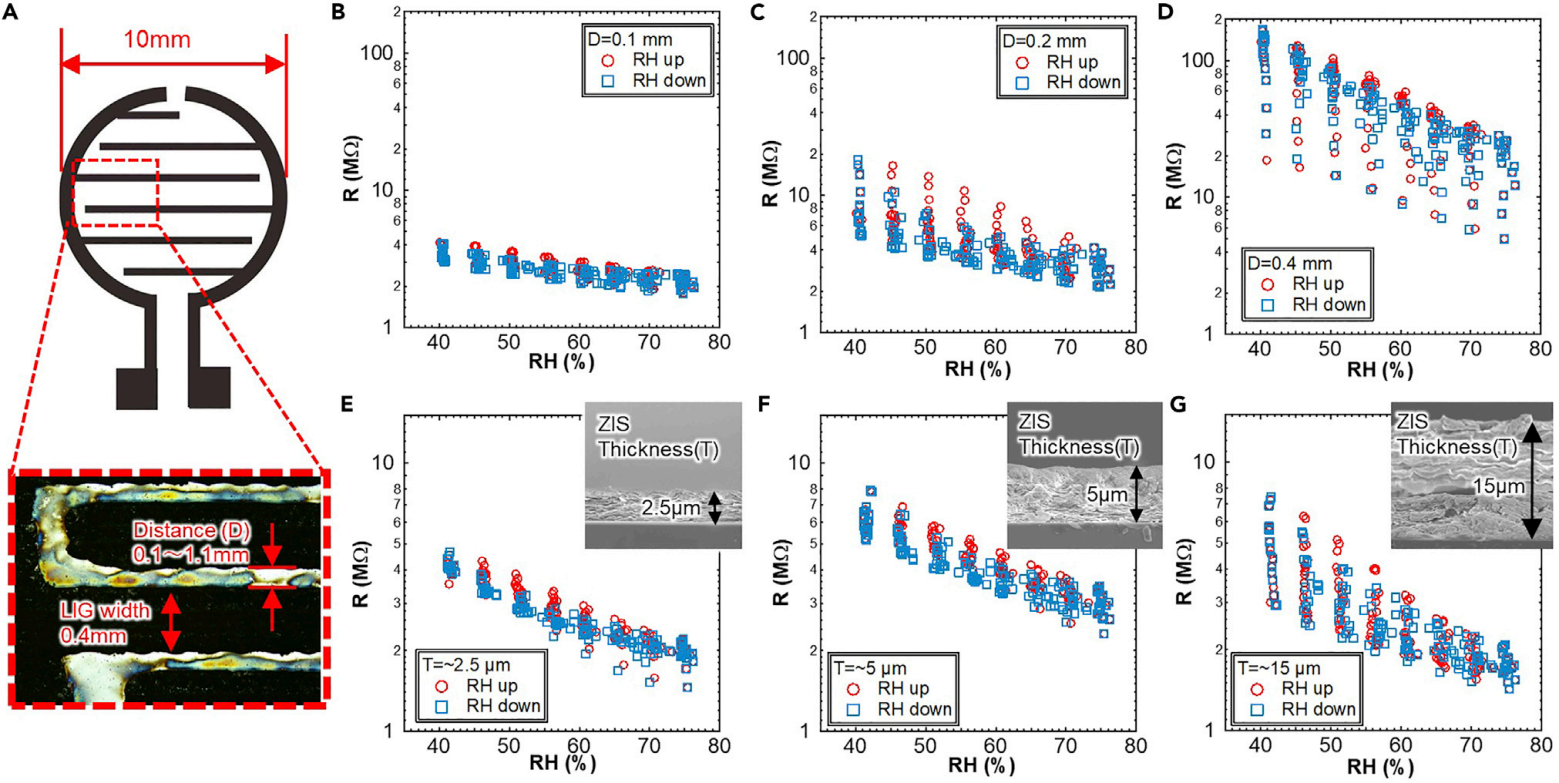
Fundamental characteristics of LIG humidity sensor at different device designs (A) LIG interdigital electrode design and definition of electrode distance and width. Multiple cycle resistance measurements of ZIS sensor at (B) *D* = 0.1 mm, (C) *D* = 0.2 mm, (D) *D* = 0.4 mm, (E) *T* = ∼2.5 μm, (F) *T* = ∼5 μm, and (G) *T* = ∼15 μm under humidity change from RH40 to 75%. RH value was also measured with a commercial humidity sensor in the oven.

Because multiple layers of ZIS are used for the humidity sensor membrane, electron paths for the resistive sensor rely on the number of interfaces between nanosheets. To analyze this dependence, we focused on the size dependence of the sensor, specifically the electrode distance and the thickness of the ZIS film. First, the electrode distance, *D*, was changed from 0.1 to 1.1 mm while the LIG width and total sensing diameter were fixed at 0.4 and 10 mm, respectively (Figure 2A). Cycle tests of humidity sensors with different *D* were conducted in a moisture- and temperature-controlled oven. The humidity was changed in RH5% increments between RH40% and RH75% with RH1%/min change ratio, and each humidity setting was maintained for 10 min at a constant temperature of 35°C. The sensor resistance extracted from 20 cycles, i.e., 75 h, was then compared to that of a commercial humidity sensor (Figures 2B–2D). The sensor resistance gradually decreased as the humidity increased from RH40% to RH75%.

To explore the stability of the sensor, hysteresis behavior was observed. Figure 2B reveals that at *D* = 0.1 mm, which is the shortest electrode distance examined, the sensor exhibits almost no hysteresis even after 20 cycles of humidity changes. By increasing the electrode distance to *D* = 1.1 mm (Figures 2C, 2D, and S1), hysteresis and base resistance increase. Based on these results, *D* = 0.1 mm is the optimal distance for the LIG interdigital electrode. It should be noted that a distance shorter than 0.1 mm may yield better stability. However, using the CO_2_ laser ablation method to form LIG on PI, as done in this study, *D* = 0.1 mm is the shortest design owing to a machine limitation. To further optimize humidity sensing behavior, the thickness dependence of the ZIS stacking layer was investigated by changing the thickness, *T*, from ∼2.5 μm to ∼15 μm at *D* = 0.1 mm. Thinner ZIS shows less hysteresis and constant resistance, even after 20 cycles (Figures 2E–2G). Based on the results for electrode distance and thickness, a shorter electrode distance (D = 0.1 mm) and thinner ZIS layer (T = ∼2.5 μm) are ideal to achieve better performance with less hysteresis and higher stability.

Based on the results about electrode distance and thickness dependences, instability is mostly caused by interfaces of ZIS nanosheets, so that a small number of interfaces, i.e. shorter distance and reduced thickness, are better to achieve long-term stability and precise monitoring of humidity.

**Figure 3:**
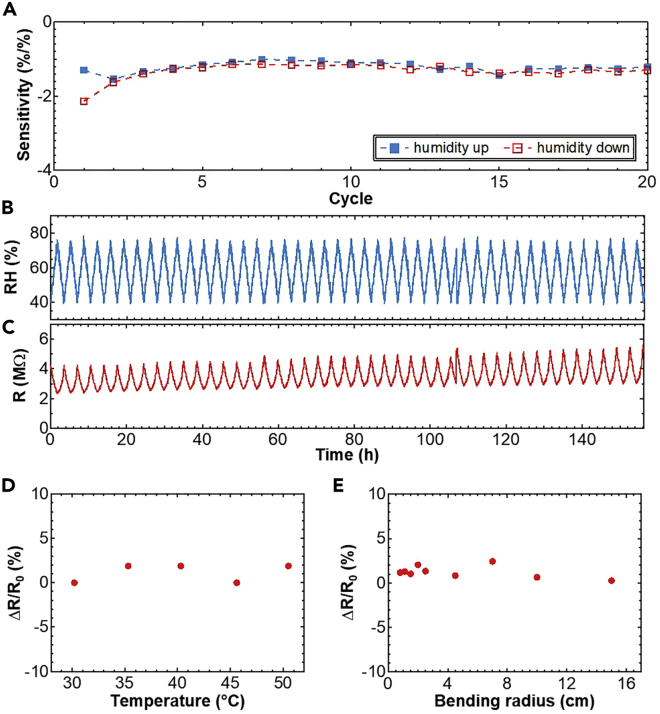
Stability properties of the LIG humidity sensor (A) Sensitivity at each cycle as humidity increases and decreases. Long-term cyclic measurements using (B) a commercial humidity sensor and (C) the optimized humidity sensor in the oven under humidity control from RH40% to 75%. (D) Temperature dependence of the humidity sensor from 30°C to 50°C at RH 40%. (e) Bending test of the flexible ZIS humidity sensor under RH 40%.

Using the optimized device design configuration, humidity sensitivity for 20 cycles, while changing the humidity from RH50% to RH70% and back again, was confirmed. The sensitivity (∼1.1%/% (resistance change ratio vs. humidity level change)) is relatively stable even after 20 cycles (Figure 3A). To ensure the durability of a ZIS humidity sensor, a long-term continuous cycle test of ∼150 h was also conducted (Figures 3B–3C). The optimized humidity sensor was more stable than our previous report of about 100 h (Lu et al., 2021a). As the sensor, response time is another important characteristic. To check the response speed to humidity, breath was intentionally applied over the sensor to quickly change humidity (Figure S2). The results show that response and recovery times are <0.8 s and <4 s, respectively. The recovery time to completely return to initial value is relatively slow compared to the response time. This is because it takes time to desorb water molecules from the surface of ZIS. However, this response is still good enough to monitor breath in a mask.

To further investigate properties of the ZIS humidity sensor, the influence of temperature and mechanical bending on the sensor were investigated in terms of their effect on the resistance change ratio, ΔR/R_0_, where ΔR is the difference between initial resistance, R_0_, at 30°C in the flat state and the set temperature or bending radius, respectively. The resistance change ratio of the ZIS humidity sensor remains essentially invariant at temperatures from 30°C to 50°C (Figure 3D). Furthermore, almost no noticeable change in resistance occurs under various bending radii up to 0.8 cm (Figure 3E). This design is most likely ideal for ZIS with LIG electrodes in terms of stability for long-term use in different environments, in which temperature, humidity, and mechanical bending often change, as in a device that is being worn.

### Demonstrations of the respiration monitoring

**Figure 4:**
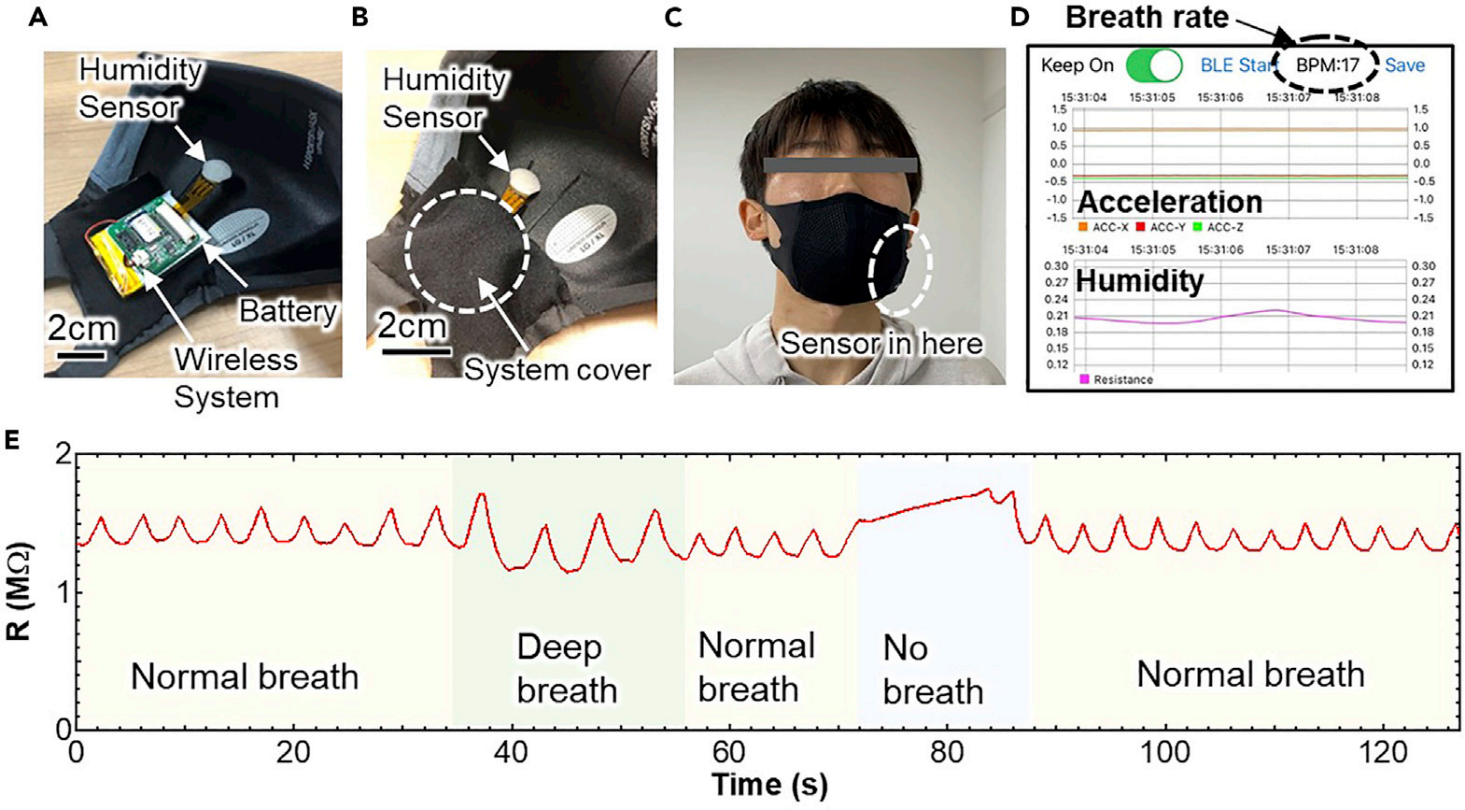
Wireless humidity sensor for breath monitoring Photos of the sensor system with humidity sensor, wireless system, and battery (A) before and (B) after putting it in the pocket. (C) Photo worn by the subject. (D) Display of App during measurement. (E) Real-time humidity sensor resistance varies in response to normal, deep, and no breathing.

To track respiration activity, a wireless ZIS humidity sensor was then developed (Figure 4A). The wearable sensor system was assembled with a circuit, a small battery, and a ZIS humidity sensor. The circuit consists of a 3-axis accelerometer, a signal processing module, and a Bluetooth wireless signal transmission module. The sensor system was placed inside a pocket on a face mask, which has a mesh structure in order not to interfere with humidity changes caused by breath (Figures 4B and 4C). Furthermore, this mesh structure helps to replace the air in the mask quickly, so that accumulation of water molecules in the mask can be prevented. Data are transmitted via Bluetooth from the system to a smartphone. To achieve real-time monitoring, an app was developed to check the movement of a subject and respiration rate on a smartphone wirelessly (Figure 4D). The breathing rate per minute (BPM) was calculated as the time interval between the peaks of humidity signals. As a volunteer breathed, the sensor detected a humidity change. Depending on the number of peaks and also the resistance, breathing conditions such as normal breaths, deep breaths, rapid breaths, coughing, and cessation of breathing were successfully monitored (Figure 4E, Video S1). For example, when the volunteer respired deeply, the atmosphere in the mask became more humid, and the resistance was accordingly lower than when breathing normally. On the other hand, when a volunteer stopped breathing, this resulted in a gradual increase of the resistance due to lower humidity in the mask. It was confirmed that the number of peaks of resistance change is well matched with the BPM.

Video S1. Demonstration of respiration monitoring using a mask, Related to Figure 4

**Figure 5:**
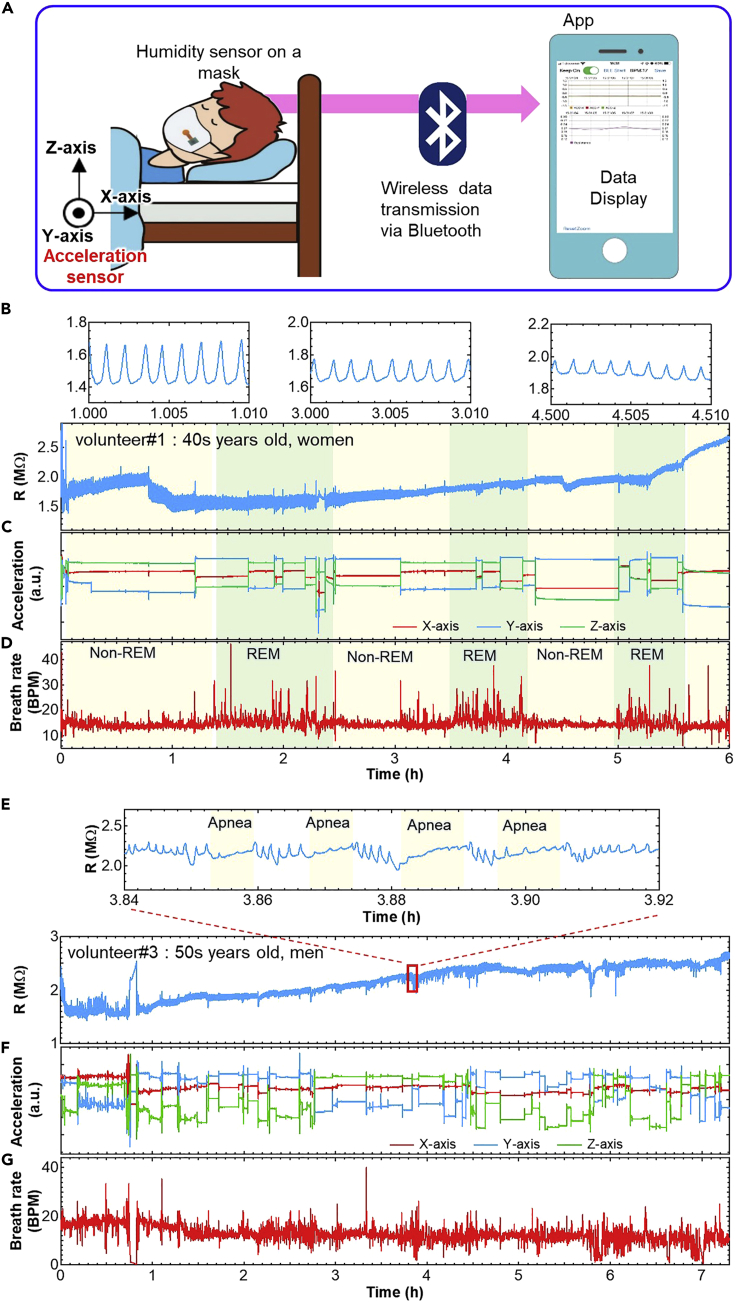
Sleep apnea monitoring results (A) Measurement setup of the system. Real-time monitoring results from volunteer #1 of (B) humidity sensor resistance, (C) three-axis acceleration sensor output, and (D) breathing rate calculated from peaks of humidity sensor output during sleep. Real-time monitoring results from volunteer #3 for (E) humidity sensor resistance, (F) three-axis acceleration sensor outputs, and (G) breathing rate calculated from peaks of the humidity sensor response during sleep.

Sleep apnea syndrome (SAS) is a breathing disorder that causes human breathing to slow or stop for brief periods during sleep. If this sensor system can monitor SAS during sleep at home, it will be useful to detect the syndrome in its early stage. A wearable wireless sensor system on a mask was employed to monitor breathing rate and movement during sleep as a proof-of-concept for home-use SAS diagnosis. Three volunteers were invited to monitor their respiration rates during sleeping. They wore the mask with the sensor system, and real-time and continuous breathing rate and body motion during sleep were recorded using a smartphone (Figure 5A). Figures 5B–5D show the monitoring results of volunteer #1, a woman in her 40s. The sensor system successfully recorded respiration rate and body motion during sleep for up to 6 h. Although there are large and small peak height differences of humidity sensor output, the ZIS resistance value during sleep was stable within the range in which the respiratory rate could be extracted (Figure 5B). In addition, three-axis acceleration sensor output indicates the volunteer’s sleeping posture and movement during sleep, which also includes information necessary to diagnose sleep quality (Figure 5C). In general, breathing rates change during sleep, indicating whether the subject was in non-REM sleep or REM sleep. For non-REM sleep, breathing rate decreased slightly compared to the rate in the awakening stage when it becomes almost constant. On the other hand, during REM sleep, the pattern had large variation with an overall increase in BPM. To confirm this behavior, the continuous BPM of the volunteer was extracted from resistance peaks (Figure 5D). Breathing rates were about 14 BPM for non-REM and large variation rate for REM sleep during periods of 60–90 min. These results are in good agreement with standard trends of REM and non-REM sleep. Furthermore, the acceleration sensor clearly shows that there was less body movement during non-REM sleep, and more during REM sleep. This is also consistent with the fact that human activity in sleep is reduced during non-REM sleep. For volunteer #1, measurement during sleep was monitored differently from humidity, in that the sensor was attached to her face without a mask. Breathing rate patterns to recognize REM and non-REM sleep were almost the same as those with the sensor on the mask (Figure S3). This suggests that the sensor system integrated in a mask can be used instead of conventional breathing monitoring, which attaches the sensor directly to the face. The sensor on a mask is more convenient and comfortable for measuring breathing rate. It should be noted that due to improvement of long-term stability of the flexible sensor, practical continuous respiration monitoring during entire sleep could be successfully demonstrated. Compared to other reports for the respiration monitoring (Table 1), this study shows more advance to realize long-term stable sleep monitoring using a flexible sensor platform wirelessly as the practical application. Furthermore, by monitoring both ZIS resistance change and acceleration sensors, coughs can be most likely distinguished (Figure S4). This result tells that this wearable sensor may be able to monitor more health conditions in addition to respiration.

**Table 1:** Comparison of the wearable respiration monitoring

Refs	Sensor	Flexible sensors	Area to measure	Wireless system	Continuous monitoring	Sleep monitoring
Zhong et al. (2021)	Pressure sensor	Yes	Mask	Yes	∼70 s	No
Zavanelli et al. (2021)	Multiple sensors	No	Sternum area	Yes	>5 h	Yes
Lu et al. (2021b)	Humidity sensor	Yes	Mask	Yes	∼12 min	No
Pang et al. (2018)	Humidity sensor	Yes	Mask	No	∼1 h	No
Xie et al. (2019)	Humidity sensor	Yes	Mask	No	∼10 min	No
Escobedo et al., 2022	CO_2_ sensor	Partially yes	Mask	Yes	∼150 min	No
This study	Humidity sensor	Yes	Mask	Yes	>7 h	Yes

Next, reproducibility of this measurement was confirmed by repeating the monitoring from this and two other volunteers. First, breathing rate and body motion during sleep could be repeatedly monitored during sleep regardless of the volunteer (Figure S5), although there was large variation for volunteer #2, a man in his 40s (Figure S5B). When BPM exceeds 25, it is most likely just noise. This noise may occur if the mask is pressed into the pillow, causing a change in humidity inside the mask due to accumulation of moisture. Based on the results from these two volunteers, no SAS behaviors were detected. To confirm SAS behaviors, volunteer #3, a man in his 50s, who has symptoms of SAS, was invited to participate. As expected, some sensor output peaks corresponding to breathing disappeared for more than 10 s (Figures 5E–5G). Apnea interferes with restful sleep and the acceleration sensor showed that volunteer #3 moved frequently (Figure 5F). Furthermore, the subject’s BPM varied widely due to reduced or halted respiration (Figure 5G). To confirm that this result was not due to a technical problem, the experiment was performed twice more. Similar trends, especially for breathing rate, were again recorded (Figure S6). These results show the utility of using this sensor system to monitor SAS at home because some peaks match the waveform of no breathing (Figure 4E) and the subject is aware that he has symptoms of SAS.

In this study, a wireless, flexible humidity sensor system for real-time monitoring of sleep respiration was developed by optimizing a humidity sensor design and successfully monitoring respiration during sleep. We found that a smaller LIG interdigital electrode distance and thinner ZIS nanosheets for humidity sensing show greater stability. After confirming the stability of the sensor, wireless, real-time breathing rate and movement were monitored in three volunteers during sleep, based upon wireless humidity sensor system on a mask. This wearable humidity sensor and a smartphone app successfully recorded respiration including normal, deep sleep, and identified symptoms of SAS in a volunteer with sleep apnea. Based on real-time BPM data from the humidity sensor, trends of REM, non-REM, and SAS symptoms were clearly monitored. Although many challenges remain to replace conventional medical SAS tools, this mask-based wireless sensor system may be a good platform to advance home-use medical and healthcare diagnostic electronics.

### Limitations of the study

In this study, we have discussed the stability factor of the stacked ZIS nanosheet film, concluding that interface between nanosheets affects the stability. However, we did not find the specific material or structure at the interface caused for an instability factor. For the physiological trial, we only showed the possibility to find sleep apnea syndrome from respiration monitoring. For the practical use, clinical trials compared to the conventional methods will be required.

## STAR★Methods

### Key resources table

**Table:** 

REAGENT or RESOURCE	SOURCE	IDENTIFIER
**Chemicals, peptides, and recombinant proteins**		

ZnCl_2_	Sigma-Aldrich	CAS 7646-85-7
InCl_3_⋅4H_2_O	Sigma-Aldrich	CAS 22519-64-8
Thioacetamide	Sigma-Aldrich	CAS 62-55-5
Ag paste	Asahi Chemi., Japan	SW-1600C

### Resource availability

#### Lead contact

Further information and requests for resources and reagents should be directed and will be fulfilled by the lead contact, Professor Kuniharu Takei (takei@pe.osakafu-u.ac.jp or takei@omu.ac.jp).

#### Materials availability

This study did not generate new datasets.

### Methods details

#### Synthesis of ZIS nanosheets

The ZIS solution was prepared from ZnCl_2_ (Sigma-Aldrich, 99.999% trace metal basis), InCl_3_·4H_2_O (Sigma-Aldrich, 97%), and thioacetamide (Sigma-Aldrich, ≥99%). The detailed process has been previously reported^9^. In this work, a ZIS nanosheet was formed using a solution of 10 mg mL^−1^.

#### Device fabrication

LIG electrodes of interdigitating pattern were formed using CO_2_ laser ablation (VLS2.30, UNIVERSAL Laser System, power: 4 W; speed: 50 cms^−1^) on a PI film (thickness: 50 μm). The width of the electrodes was 0.4 mm, and the distance between interdigitating electrodes was varied from 0.1 mm to 1.1 mm in order to find the optimal distance. Subsequently, 75 μL of the ZIS nanosheet solution was drop-cast and annealed at 100°C for 20 min. To connect this humidity sensor to a measurement system, silver (Ag) electrodes were printed with a dispensing machine and annealed at 70°C for 30 min. Finally, the surface of the humidity sensor was covered with a humidity pass filter (TEMISH, Nitto Denko) to protect the ZIS nanosheet film from contact with water.

#### Characterization

Fundamental experiments with the ZIS humidity sensor were performed in a humidity and temperature controllable oven (Espec, SH-222). All were recorded with a multi-channel data logger (Hioki, LR8400). As controls, commercial humidity and temperature sensors were also used to check humidity and temperature in the oven.

#### Real-time monitoring of sleep respiration

The humidity sensor with a wireless circuit board was placed in a mask pocket and worn by volunteers (2 males and 1 female) during sleep. Humidity and acceleration sensor outputs were automatically recorded by a smartphone linked via Bluetooth. Human subject experiments were performed in compliance with a protocol approved by the ethics committee at Osaka Prefecture University. Informed consent was obtained from all volunteers to record and use all data.

### Quantification and statistical analysis

Our study does not include statistical analysis or quantification.

## Data Availability

• Original data reported in this paper will be shared by the lead contact upon request. • This paper does not report original code. • Any additional information required to reanalyze the data reported in this paper is available from the lead contact upon request.
