# Supplementary material for: A wearable, flexible sensor for real-time, home monitoring of sleep apnea
Source: iScience. 2022 Mar 25;25(4):104163. doi: 10.1016/j.isci.2022.104163 (PMC9010767; doi:10.1016/j.isci.2022.104163)
Supplement: Supplementary file 2 — Document S1. Figures S1–S6 [file mmc1.pdf]

iScience, Volume *25*

Supplemental information

A wearable, flexible sensor for real-time, home monitoring of sleep apnea

Satoko Honda, Hyuga Hara, Takayuki Arie, Seiji Akita, and Kuniharu Takei

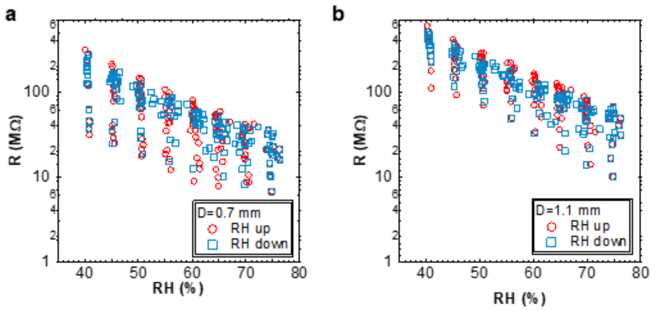

Figure S1. Fundamental characteristics of LIG humidity sensor at different device size, Related to Figure 2. ZIS humidity sensor resistance at (a) D=0.7 mm and (b) D=1.1 mm of LIG electrode distance under humidity change from RH40 to 75%.

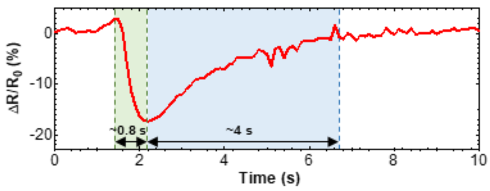

Figure S2. Response time of ZIS humidity sensor by applying breath over the sensor, Related to Figure 3.

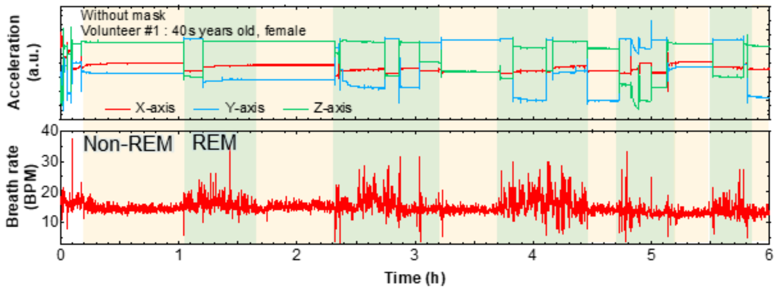

Figure S3. Long-time breath monitoring results during sleep, Related to Figure 5. Real-time monitoring results of (a) three-axis acceleration sensor outputs and (b) BPM calculated from humidity resistance peaks by attaching the sensor directly to volunteer #1's face during sleep.

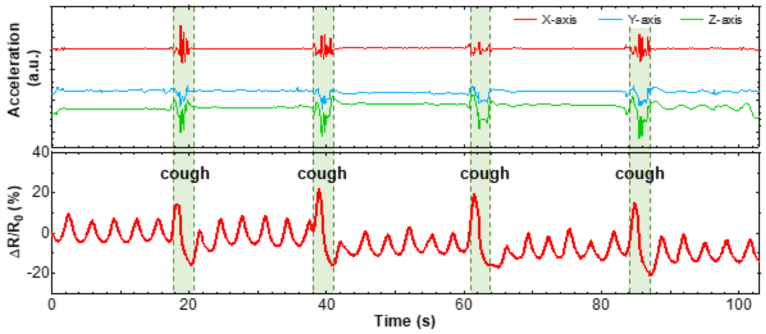

Figure S4. Real-time monitoring of acceleration sensor and humidity sensor with coughs, Related to Figure 5.

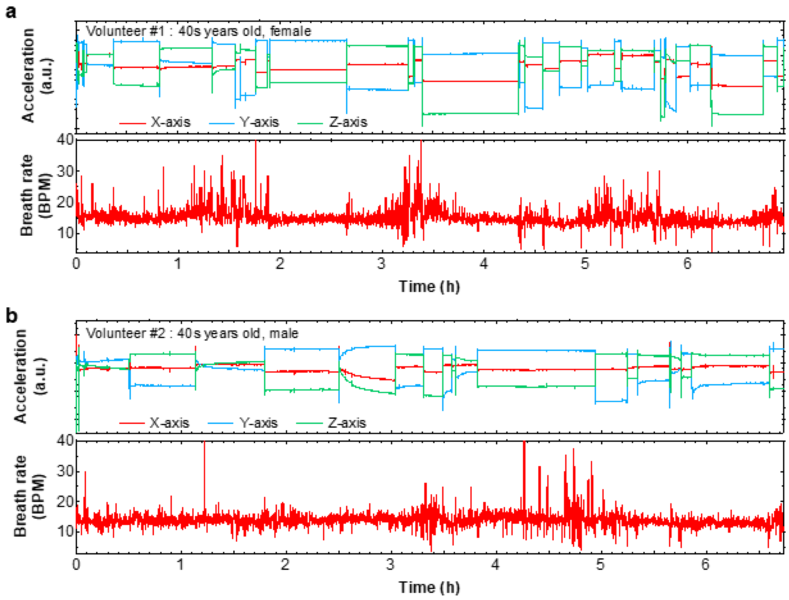

Figure S5. Real-time sleep apnea monitoring, Related to Figure 5. Real-time monitoring results of three-axis acceleration sensor output and BPM from (a) volunteer #1 and (b) volunteer #2.

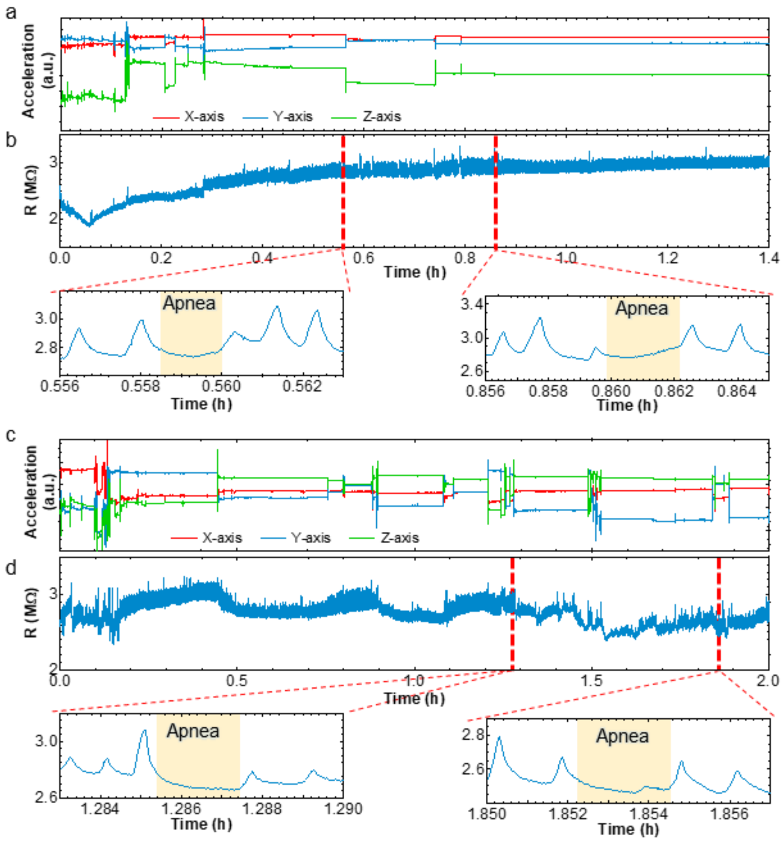

Figure S6. Real-time sleep apnea and specific apnea results, Related to Figure 5. Real-time monitoring results recorded from volunteer #3 of (a and c) three-axis acceleration sensor outputs and (b and d) humidity sensor resistance measured on different nights during sleep.
